# Transition towards net zero emissions: Integration of a PV/T system with a hydroelectric generator and the impact of demand-side management

**DOI:** 10.1016/j.heliyon.2024.e37099

**Published:** 2024-08-28

**Authors:** Armel Zambou Kenfack, Modeste Kameni Nematchoua, Elie Simo, Ghislain Junior Bangoup Ntegmi, Venant Sorel Chara-Dackou

**Affiliations:** a Energy and Environment Laboratory, Department of Physics, Faculty of Science, University of Yaoundé I, PO Box 812, Cameroon; b Carnot Energy Laboratory (CEL), Department of Physics, Faculty of Science, University of Bangui, PO Box 1450, Bangui, Central African Republic

**Keywords:** Energy transition, Net zero emissions, PV/T system, Hydroelectric generator, Demand management

## Abstract

With the aim of diversifying different energy sources and achieving net zero emissions, hybrid renewable energy sources (HRES) represent the future of the world. However, several HRES simulation software do not integrate the Photovoltaic/thermal (PV/T) system. This article designs an optimal design model for a tri-hybrid Photovoltaic/thermal/hydroelectric (PV/T/H) system for a rural locality in the North Cameroon region. The two Demand Side Management (DSM) strategies used reveal that the DSM strategy significantly reduced the energy cost by 59 % and the emission by ${\text{CO}}_{2}$ 22 % compared to the No-DSM mode. Although the use of battery storage (BSS) is used in both cases, the optimal solutions obtained thanks to the multi-objective optimization method implemented on Matlab led to 418 PV/T panels, 2 MH generators, 2 diesel generators (DG) and 217 PV/T panels and 1 DG for DSM and No-DSM mode respectively. This study is a demonstration of the effect of dynamic tariffs and active demand management technologies on PV/T/H modeling and optimization. It also reveals the need to hybridize PV/T with other energy systems to increase performance and achieve net zero emissions.

## 1 Introduction

Electrification of rural areas is a major challenge in many developing countries. Traditional solutions based on diesel generators suffer from high costs and a significant environmental impact. Renewable energies are undoubtedly alternatives to fill the gaps caused by fossil fuels. However, within the different solutions, there are many shortcomings such as the intermittency of solar energy and the dependence on rivers for hydroelectricity [1,2]. Research is therefore being pushed further towards the development of hydroelectric-solar hybrid technologies. However, their design and optimal management remain a challenge. In addition to the diversification of energy sources, hybridization allows production to be stable during summer periods and heavy precipitation [3,4]. The use of the system can be either autonomous with a large storage capacity or connected to the network to either feed the excess into the network or draw from the network to satisfy the load [5,6]. In the literature, previous studies explore several demand management systems (DSM) based on HRES system (Hybrid renewable energy system). Djeudjo et al. [7] developed a DSM model consisting of three HRES including photovoltaics, hydroelectricity and wind power for residential, commercial, hospital and educational loads in several sub-Saharan areas. Their studies reveal that HRES systems make it possible to avoid 46.5 times the GHG emissions of the diesel generator with 198 PV (photovoltaic) panels, 10 wind turbines and a hydroelectric and diesel generator for a COE (cost of energy) of 0.096 $/kWh using a MOPSO method (multiobjective particle swarm optimization). Likewise, the authors [8] designed an identical model by obtaining a minimum COE of 0.21$/kWh for residential buildings by maximizing the renewable fraction with the PV-battery system. The authors [9] developed a load management method in several rural areas using hydro-solar- wind HRES. The model led to minimal energy cost. But previous studies do not present enough variables influencing the system. Software such as HOMER, PVSYST or Retscreen have also been shown to be very effective in analyzing the feasibility of projects at HRES. But the absence of data and even more real data reduces the accuracy of the sizing. Extensive studies [10,11] on small head hydropower plants show that they have lower capital costs, which could justify the low COE. The authors [12] carried out a numerical study of a mini-hydroelectric power station allowing us to know the different costs of the system according to the design parameters using genetic algorithms. Their studies reveal the feasibility of the system with a capacity of 6.32 MW with an LCOE of $0.05/kWh. Among other things, the authors [13,14] reveal in their studies that the amount of energy that the machine can produce without interruption throughout the year, generally disagrees with the theoretical nominal power when evaluated in using different turbines while maintaining the same flow rate and turbine efficiency values. With a water height of 37 m and a flow rate of 2.97 $m^{3}/$ s, a production of 40958 MWh/year of energy produced is estimated with turbine 1 Jet Pelton. Sometimes also very neglected in research work, the length of penstocks which are pipes which transport water under pressure from the water intake to the turbine of the hydroelectric power station. Generally, in small hydroelectric power plants, this length of penstocks can be from a few hundred meters to a few kilometers [15]. In the design of HRES models, the Diesel generator is often used as a backup power source. It thus helps to fill the production deficit, although increasing the index of polluting gases in the atmosphere [16]. Additionally, this may result in underutilization of DG during periods of low demand. The study carried out by Irshad et al. [17] demonstrates that it is capable of satisfying the load using a PV/Wind/Hydro HRES system without a battery storage system (BSS). This significantly reduced the COE of the system to $0.35/kWh. Although in contradiction with the authors [18,19] who show that the reliability of an HRES system is higher when there is connection with a BSS. But the use of a battery storage system can also influence the optimal size of the DG. Batteries can help compensate for fluctuations in solar production and manage peak load, reducing the power requirements of the diesel generator. An experimental study done by the authors [20] concluded that to guarantee the reliability of a solar PV/diesel hybrid system, it is recommended to have a diesel generator whose rated power corresponds to the peak load.

From the literature, it is seen that existing studies have mainly focused on techno-economic modeling and configuration optimization of hybrid system components. However, detailed analysis of the impact of different DSM strategies on overall performance remains limited. In addition, most work is based on simplified hypotheses concerning user loads and consumption profiles. In addition to the fact that few case studies on HRES in sub-Saharan Africa exist in the literature, the observation made is.

- That there is no real optimal design model for an HRES including the solar thermal collector;
- Most studies on HRES do not take into account the DSM strategy and its impact on overall performance;
- studies consider the electric charge stable, yet its fluctuation has an effect on the HRES sizing;
- The fluctuation of the HRES input variables are taken constant in the HRES models;
- Several studies are carried out with the Homer software which sometimes leads to oversizing of the system;
- The limitation of the Homer software due to the no-implementation of the PV/T system in the simulation.

This research work therefore aims to propose an optimal design model for a tri-hybrid PV/T/Hydro solar system.

Its main contributions are.

- An optimal design model of a tri-hybrid photovoltaic/thermal/hydroelectric system accommodated with a battery and a diesel generator is developed for a rural area in North Cameroon;
- The values of cost of energy (COE), emission rate (TE), load fraction (LPSP) and renewable factor (RF) are quantified;
- The effect of each demand management strategy on the design of an HRES is presented;
- The multi-objective optimization (MOO) method makes it possible to identify the optimal output parameters (number of panels, hydroelectric generators and days of autonomy);
- The energy contributions of each system are analyzed and quantified in the area;
- Concrete recommendations to decision-makers and stakeholders are presented.

This article is presented in three main sections, so this introduction is section 1, followed by section 2 entitled methodology, section 3 entitled results and then discussion of section 4 which concludes the present study.

## 2 Methodology

### 2.1 Description of the physical model

**Fig. 1:**
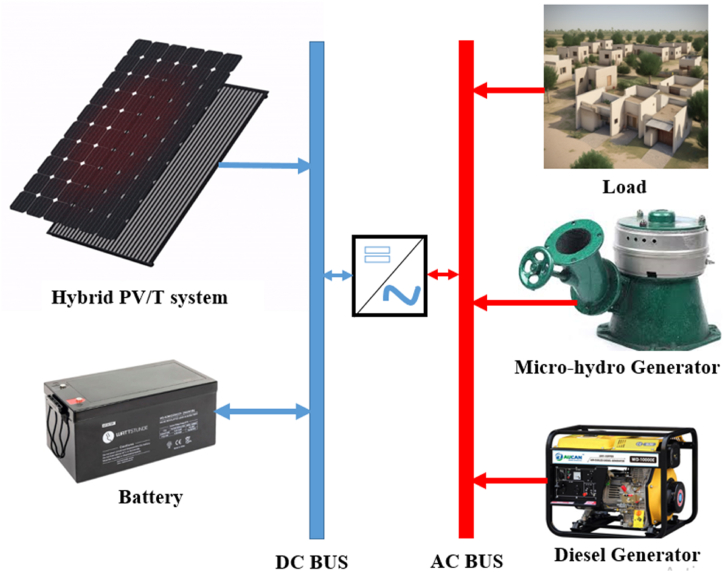
Bogo ’s electrical load.

As shown in Fig. 1, the device is made up like HRES of a PV/T system and a micro-hydro-generator. In addition, an electric storage battery and a diesel generator. Each component is represented by its power which is modeled in the following parts.

The use of a hybrid system comprising both a PV/T system, a micro-hydro-generator, an electric storage battery and a diesel generator has several advantages and makes it possible to optimize energy production in different conditions. As an advantage of such a power management system, there are.

- Diversification of energy sources: By combining a PV/T tri-hybrid system and a micro-hydro generator, the hybrid system can take advantage of the advantages of both renewable energy sources. The PV/T system works well under sufficient sunlight, while the micro-hydro generator can produce power when water is available. This allows diversification of energy sources, thus reducing dependence on a single energy source;
- Complementarity of energy sources: Solar and hydraulic energy sources have complementary characteristics in terms of availability. For example, solar power is available during the day when sunlight is at its peak, while hydro power can be more consistent and predictable throughout the year. By combining these sources, the hybrid system can better respond to seasonal and diurnal variations in energy availability;
- Management of variability and intermittency: The integration of an electric storage battery in the hybrid system makes it possible to store excess energy produced by PV/T and micro-hydro sources and release it when demand exceeds production. This helps mitigate the inherent variability of renewable energy sources and provide more stable and reliable power.
- Reduction of emissions and dependence on fossil fuels: By integrating renewable energy sources such as PV/T and micro-hydro-generator, as well as battery storage, the hybrid system helps reduce energy consumption. fossil fuels and associated greenhouse gas emissions. Diesel generator use can be limited to periods when other energy sources are not sufficient, thus contributing to a reduced carbon footprint;
- Flexibility and autonomy: The addition of a diesel generator in the hybrid system offers a backup or supplemental solution in the event of prolonged periods of low availability of renewable energy sources or high energy needs. This gives greater flexibility and autonomy to the system, ensuring a continuous supply of energy, even in adverse conditions. But for its broadcast, it is preferable to have a system which satisfies the demand without resorting to the CEO.

The physical model of the HRES developed here is based on mathematical equations describing the system. Thus the following hypotheses are established.

### 2.2 Modeling of the PV/T panel

The power of a thermal photovoltaic (PV/T) panel can be modeled by considering the photovoltaic and thermal components of the system. Strongly depending on meteorological data of solar irradiation G (W/$m^{2}$) and ambient temperature, the output power of PV/T is given by equation (1) [21].

(1) $$P_{PV/T}=P_{PV}+0.38\times P_{T}$$

with $P_{PV}$ the electrical power of the PV and $P_{T}$ the thermal power of the thermal collector below PV given respectively by equations (2), (3) [9,22]. The term 0.38 represents the conversion coefficient of the equivalent thermal energy into electrical energy produced by the system.

(2) $${P_{PV}=f}_{v}\times P_{r}\times \left(\frac{G}{G_{ref}}\right)\times \left(1+K_{temp}\left(T_{C}-T_{ref}\right)\right)$$

(3) $$P_{T}=A\times {ղ}_{th}\times G\times \left(T_{C}-T_{a}\right)$$

The terms $f_{v}$, $P_{r}$, G, $K_{temp}$, A and ${ղ}_{th}$ denote respectively the derating factor, the nominal power, the solar radiation, the surface area of the PV and the thermal efficiency and therefore the values are available in Table 1. The temperature of the PV cells is expressed through equation (4) [21[23]].

(4) $$T_{c}=T_{a}+\left(0.0256\times G\right)$$

**Table 1:** Input data [22,24].

$f_{r}$	**0.9**	$T_{ref}$	**25°C**
$G_{ref}$	**1000**	${ղ}_{tu}$	**0.85**
$K_{temp}$	−**4.1**$\times 10^{-3}/{}^{\circ}C$	${ղ}_{g}$	**0.9**
${ղ}_{th}$	**0.75**	ρ	**1000kg/**$m^{3}$
**A**	**1.3**$m^{2}$	$P_{r\_dg}$	**4 kW**
**g**	**9.81 N/kg**	μ	**0.246**

### 2.3 Modeling of the MHG

The power of the MHG is strongly dependent on the flow rate Q ($m^{3}$/s), the head of water $H_{n}$**, and** the efficiency of the turbines ${ղ}_{tu}$ and the generator ${ղ}_{g}$ as expressed in equation (5) [9,10,14]. It should be noted that this simplified modeling does not account for additional losses and inefficiencies, such as friction losses, turbulence losses, or electrical losses.

(5) $$P_{MHG}=\left\{ \begin{array}{c} Q\times \rho \times g\times H_{n}\times {ղ}_{g}\times {ղ}_{tu},for\;Q_{\min}\leq Q\leq Q_{\max}\\ Q\times \rho \times g\times H_{n}\times {ղ}_{g}\times {ղ}_{tu},for\;Q\geq Q_{\max}\\ 0,for\;Q<Q_{\max} \end{array}\right.$$

### 2.4 BSS modeling

Battery storage capacity is an essential characteristic to model. It represents the amount of energy that the battery can store and release. It is generally expressed in kilowatt hours (kWh) or megawatt hours (MWh). When HRES sources are in operation, it is possible that the energy is higher than the demand. In this case, excess energy is stored in the battery storage device. It thus makes it possible to avoid the provision of another source of energy during hours of high demand. Its power is expressed in equation (6) [24].

(6) $$P_{bss}=\frac{E_{L}\times AD}{{ղ}_{inv}\times {ղ}_{bss}\times DOD}$$

with the terms $E_{L}$, AD, DOD, ${ղ}_{inv}$ and ${ղ}_{bss}$ respectively representing the average energy load, the number of days of autonomy, the depth of discharge, the efficiency of the inverter and the battery [9]. Their values are presented in Table 1.

### 2.5 DG modeling

The power of the DG depends on its efficiency and the type and consumption of fuel $F_{dg}$ used. The efficiency of a DG refers to its energy efficiency, that is, the amount of electrical energy produced in relation to the energy contained in the fuel. A more efficient DG will convert more of the fuel energy into electricity, resulting in higher power output for the same amount of fuel consumed. It is expressed in equation (7) [24].

(7) $$P_{dg}=F_{dg}-\;\mu P_{r\_dg}$$

The economic parameters of this study are present in ref [7,8,25].

### 2.6 Principle of management strategies

Two management strategies are applied in the implementation of code through equations in MATLAB software. This is DSM mode (Demand Side Management) and no-DSM mode (No Demand Side Management) which are two different approaches used in household power management.

#### 2.6.1 DSM mode

**Fig. 2:**
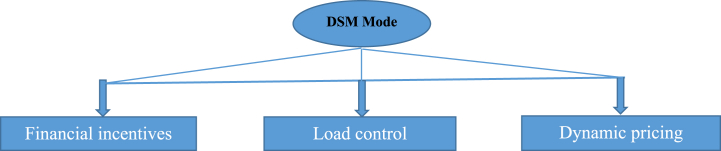
DSM mode characteristics.

DSM mode is an approach that aims to manage and modulate consumer energy demand in order to optimize the use of available energy and improve energy efficiency. The principle of DSM is based on the modification of household consumption habits and the flexibility of demand to adapt to variations in energy supply. The overall objective of DSM is to optimize energy demand, mitigate peak demand, reduce energy costs, and improve power grid efficiency [26]. The main features of DSM mode are shown in Fig. 2. Dynamic pricing is one of the methods used for this strategy. It consists of varying the COE according to the availability of energy. Higher rates can be applied during peak periods, incentivizing consumers to reduce their consumption during these periods. Among other things, a load control device via smart meters can be used to adjust devices so that loads are high outside peak hours. Financial incentives for consumers aimed at pushing them to reduce consumption during points hours in return for rewards are very effective.

#### 2.6.2 No-DSM mode

**Fig. 3:**
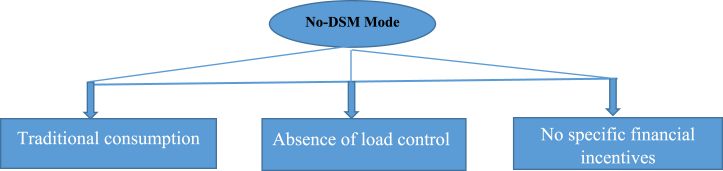
No-DSM mode features.

Non-DSM mode refers to an approach that does not implement specific demand management measures in the supply of energy to households. In this mode, household energy consumption is not modulated or controlled according to variations in energy supply. The main characteristics of the no-DSM mode are presented in Fig. 3. It is generally the most used because it implies free consumption [27].

Meteorological characteristics as well as the different devices and their nominal powers in the Bogo area are available at ref. [7]**.**

### 2.7 Principles of energy source management

The different management flows of a hybrid system using renewable energy sources and an energy storage system demonstrate the flexibility of energy management. Here is how the electrical power flow is handled in these cases.

- Case 1: The energy produced by renewable sources is sufficient to meet energy demand, and any excess is stored in the BSS.
- Case 2: In this configuration, similar to the previous one, the excess energy generated by renewable sources exceeds the storage capacity of the BSS. In this case, the excess can be sold to the electricity grid if it is close to the HRES.
- Case 3: Renewable resources cannot provide enough energy to satisfy demand. In this case, the production deficit is filled by using the energy stored in the BSS.
- Case 4: The energy from renewable sources is not sufficient to meet the energy demand, and the BSS is also discharged. In this situation, a diesel generator is activated to power the load.

**Fig. 4:**
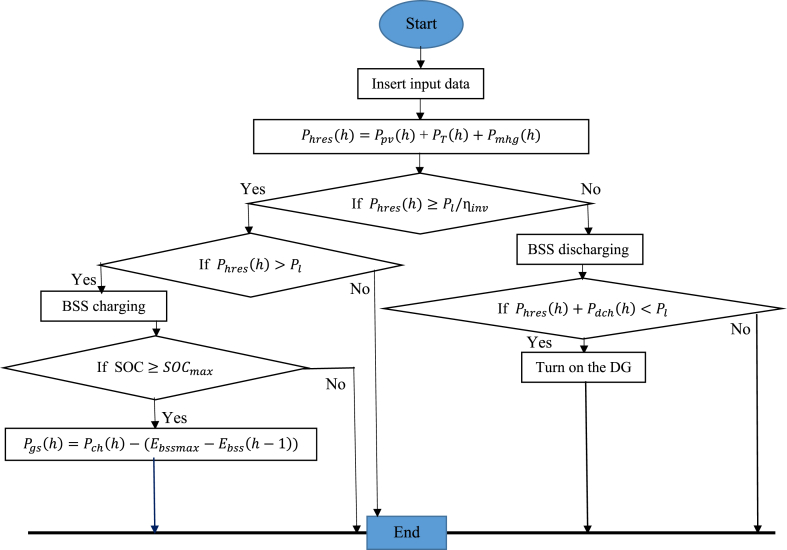
Energy flow management diagram.

**Fig. 5:**
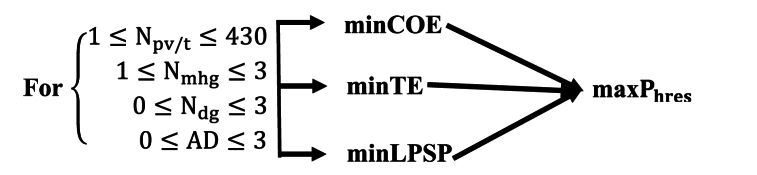
Formulation of the optimization process.

These different deadlines illustrate the flexibility of energy management in hybrid systems. By optimizing the use of available renewable energy sources and relying on energy storage and diesel generators when needed, these systems can efficiently meet energy demand while minimizing costs and reducing impact. on the environment. The energy flow diagram of these energy management strategies is shown in Fig. 4. The formulation of the optimization problem is visualized in Fig. 5.

By optimizing the use of available renewable resources, the system can meet energy demand while minimizing costs and reducing environmental impact. Additionally, using the diesel generator as a backup power source helps ensure continuous power supply in the event of a shortage of renewable energy. This demonstrates the importance of planning and efficient energy management in hybrid systems to ensure reliable and sustainable power supply. The input data for the different HRES parameters used here are available in Ref [24]**.**

### 2.8 Optimization process

The optimization process here is the multi-objective method. It consists of finding the best compromise between several competing objectives. In the context of optimizing an HRES, typical objectives include maximizing energy production, minimizing costs, reducing greenhouse gas emissions, or improving system reliability. system. Rather than looking for a single optimal solution [21]. In this context, multi-objective optimization requires defining the specific objectives to be achieved. This is to maximize renewable energy production, minimize operating costs and reduce emissions ${CO}_{2}$. Additionally, constraints can be set, such as battery storage capacity or diesel generator power limits [22] (Fig. 5).

Thus, it consists of minimizing the COE, the TE and the LPSP so that the energy production is maximum.

## 3 Results and discussion

**Fig. 6:**
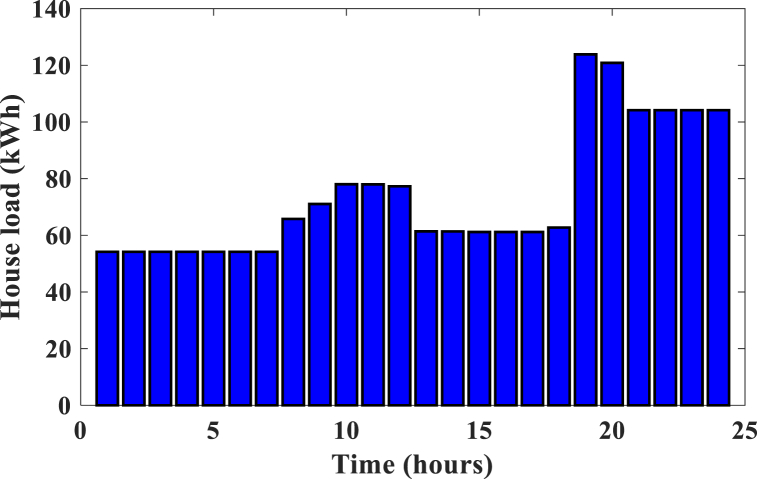
Household load profile without demand management.

**Fig. 7:**
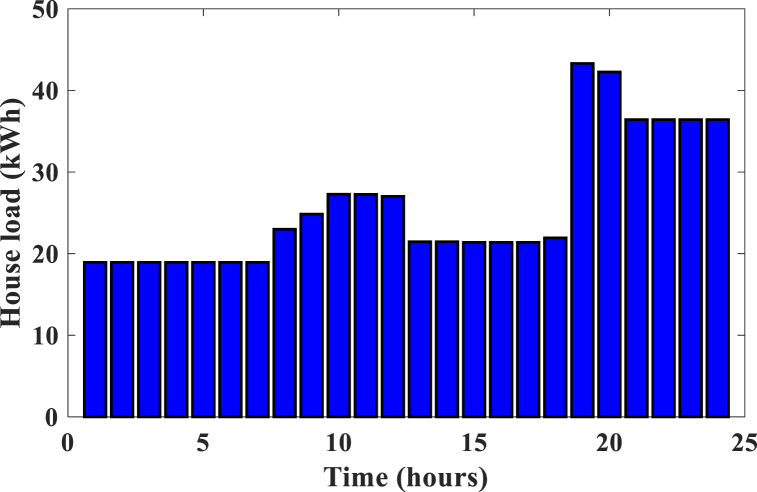
Household load profile with demand management.

This section presents the different optimal design results of the HRES for a rural area in North Cameroon. The sample was limited to 300 residential households with almost similar consumption behaviors. Fig. 6, Fig. 7 present the daily variations in loads for the two management strategies (respectively no-DSM and DSM. As a DSM method, we have the Installation of LEDs and high-efficiency electrical appliances in households and businesses. Dynamic pricing incentives (peak/off-peak rates, peak rates). Then, controlled load shedding programs in the event of peak consumption. It is theoretically three times higher when no strategy is applied. For example between 8 a.m. and 12 p.m. and 7 p.m.-midnight, correspond to peak hours and the maximum load is 134 kWh in no-DSM mode and 44 kWh in DSM mode. and improve the use of available energy resources This phenomenon can be explained by the effect of demand management on energy consumption. By applying a demand management strategy, users are encouraged to. adjust their electricity consumption during peak periods, reducing overall demand on the system. Thus, the maximum load is reduced, allowing more efficient use of available energy resources.

**Fig. 8:**
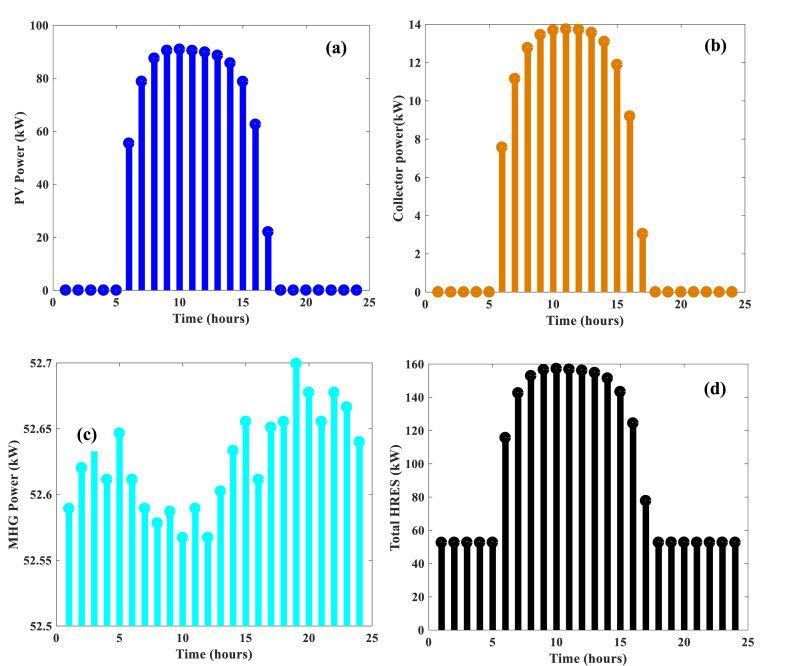
Power generated by a) PV, b) Thermal collector, c) MHG, d) Total HRES.

Fig. 8 shows the different powers generated by the tri-hybrid system. It is strongly dependent on weather conditions. For solar energy, the PV/T hybrid system in Fig. 8a and Fig. 8b look the same. PV production reaches a maximum electrical power of 94 kWh/day and that of the thermal collector reaches a maximum equivalent electrical power of 14 kWh/day. PV/T hybridization therefore increases the production of the system. In Fig. 8c, the variation of the electrical power supplied by the micro-hydroelectric generator is presented. It fluctuates between 52.5 and 52.7 kWh/day. This is justified by the flow which varies very slightly during the day, in turn affecting production very little. This is best seen in Fig. 8d which shows the total power of the tri-hybrid system. Between 00h-5h and 18h–00h, the power produced appears constant, being totally dominated during these hours by the production of the MHG in operation. It is remarkable to observe that the hybridization of several energy sources increases the production of the system.

**Fig. 9:**
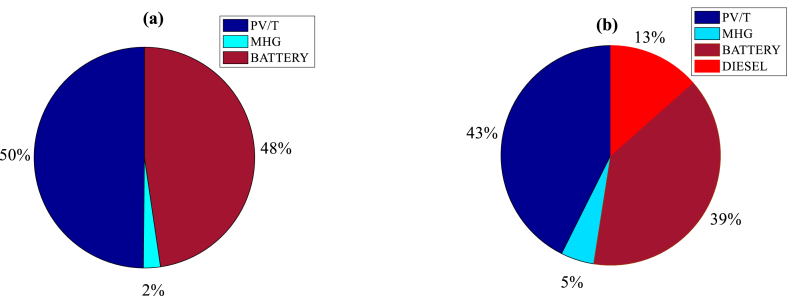
Energy contribution in mode a) DSM and b) no DSM.

The energy contributions for each management strategy are evaluated in Fig. 9a and Fig. 9b. With a DSM strategy applied in Bogo aimed at meeting demand, PV/T hybridization contributes 50 %, compared to 48 % for MHG and 2 % for the BSS system. On the other hand, without a management strategy, the PV/T contributes to 43 %, the MHG to 5 %, the BSS system to 39 % and the DG to 13 %. Thus, the DSM strategy contributes to improving the supply conditions of the electricity network. This assertion is further justified in Table 2 which presents the optimal parameters for different strategies and solutions. It reveals that the DSM strategy is better and avoids a COE of more than 59 % compared to the no-DSM mode. Likewise, the emission rate is reduced to 22 % with this strategy. A particular observation on the probability of loss of electrical power makes it possible to show that the load is most satisfied with the DSM strategy. In addition, its renewable fraction is the best. Although the addition of batteries entails an additional initial investment cost, their benefits translate into a reduction in long-term operating costs and a marked improvement in the continuity of electricity supply, reducing power outages and ensuring better quality of service for users. This increased reliability is a key factor for the socio-economic development of rural areas. Overall, battery storage proves to be an essential component of the proposed tri-hybrid system, despite its initial cost, thanks to the many benefits it brings in terms of production and demand management.

**Table 2:** Optimal output parameters.

Strategy	Solution	$N_{PV/T}$	$N_{GD}$	AD (days)	$N_{MHG}$	TE (kg/years)	LPSP (%)	COE ($/kWh)	RF
DSM	**PV/T-GH-BSS**	**217**	**//**	**1.2**	**1**	**9956.871**	**0.000951**	**0.0751**	**1**
No DSM	**PV/T-GH-BSS-Diesel**	**418**	**1**	**2**	**2**	**12753.492**	**0.0668**	**0.186**	**0.877**
**Relative deviation (%)**	**-**	**48.08**	**-**	**40**	**50**	**22**	**98**	**59**	**14**

Rural areas of northern Cameroon are often far from main national electricity networks. A decentralized hybrid PV/T/hydro system ensures autonomous electrification of these isolated localities. The low population density in these rural areas makes centralized electricity networks economically unviable. A smaller-scale hybrid system, adapted to local needs, is therefore more relevant.

Especially with limited access to fossil fuels. Providing fuel (diesel, gasoline) can be difficult and expensive in these remote rural areas. Hybridization with renewable sources makes it possible to reduce dependence on fossil fuels.

Table 3 presents the comparison of the present work with some works in the literature. The comparison of the COE obtained in this study with those from the literature shows a better, more economical and more accessible value for a rural community.

**Table 3:** Comparison with work from the literature.

Reference	HRES	Country	Methods	COE ($/kWh)
Present work	PV/T-GH-BSS-DG	Cameroon	MOO	0.186
Present work	PV/T-GH-BSS	Cameroon	MOO	0.0751
[9]	PV/WT/MHG/BSS/DG	Cameroon	MOPSO	0.096
[28]	PV/DG/BSS	Algeria	PSO	0.37
[29]	PV/WT/DG/BSS	Saudi Arabia	MOSaDE	0.083
[30]	PV/DG/BSS	Benign	HOMER	0.207
[31]	PV/WT/DG/BSS	Morocco	LARO	0.11
[32]	PV/WT/BSS/FC/DG	Chad	MOPSO	0.2982
[33]	PV/WT/BSS/DG	Cameroon	TLBO	0.2419
[34]	PV/MHG/BSS	Cameroon	HOMER PRO	0.0453
[34]	PV/MHG/BSS	Cameroon	GA	0.0344
[35]	PV/WT/FC/Electrolyzer/HSS/BSS	India	HOMER	0.232

## 4 Conclusion

This research article aims to provide an optimal demand management strategy in a rural area of Northern Cameroon. It appears that the use of a hybrid system comprising a PV/T system, a micro-hydro-generator, an electric storage battery and a diesel generator offers a balanced solution to maximize the production of renewable energy, mitigate the variability, provide reliable power and reduce overall environmental impact. Tri-hybrid hybridization increases system performance and contributes to the diversification of renewable energy sources. It contributes with the application of the DSM strategy to a reduction of 22 % in the emission rate ${\text{CO}}_{2}$ and 59 % in the cost of energy in the rural area of BOGO. Comparison of the COE with those in the literature indicates a value (0.0751$/kWh) more accessible for rural communities. This is a great contribution to the advent of net zero emissions. Compared to other software tools, MATLAB has the advantage of being a very complete platform and well suited to multi-objective optimization problems in the energy field. Its programming environment and its interaction capabilities with other software, allowing the integration of specialized models such as PV/T, make it a relevant choice for this type of complex study. These results demonstrate that the application of the DSM strategy makes it possible to optimize the use of the different energy sources of the tri-hybrid system, favoring a greater contribution from renewable sources, such as PV/T and MHG. Implementation of incentive programs for energy efficiency and self-consumption. As a recommendation, the use of dynamic tariffs and active demand management technologies should be regulated. Local stakeholders (managers, technicians) must be trained in the operation and maintenance of hybrid systems. Then raise awareness among rural communities about the issues of energy efficiency and self-consumption. The present study would be effective in decision-making and could be generalized to other rural or rural areas. However, the system did not take into account general costs such as component costs for converting thermal energy from PV/T to electricity. The study focuses only on a rural area in northern Cameroon. To improve the transferability of the model, it would be important to apply it to other geographic and climatic contexts. In addition, it would be wise to include in future work, energy from biomass which has very great potential in rural areas.

## Funding statement

This research did not receive any specific grant from funding agencies in the public, commercial, or not-for-profit sectors.

## Data availability statement

We have included the data sources in the manuscript.

**Table:** Nomenclature

A	Surface du collecteur
AD	Autonomy day
BSS	Battery
DG	Diesel generator
FC	Fuel Cell
G	Irradiation solaire
GA	genetic algorithm
H	Générateur Hydroélectrique
h	hours
HSS	Hydrogen storage system
K	Coefficient de température (/°C)
LARO	Leader Artificial Rabbits Optimization
LPSP	Loss of power supply probability
MHG	Micro hydro generator
MOSaDE	Multi-objective Self-Adaptive Differential Evolution
P	Puissance
PSO	Particle swarm optimization
PV/T	Photovoltaic/Thermal
Q	Débit ($m^{3}/s$)
SOC	State of charge
T	Température de la cellule
TLBO	Teaching-learning-based optimization
WT	Wind turbune
Greek symbols	
ղ	Efficiency
ρ	Densité de l'eau
Subscripts	
a	ambient
BSS	battery storage system
c	cell
ch	charge
dch	Dump
dg	Diesel generator
g	generator
l	load
pv	Photovoltaic
r	nominal
ref	Reférence
T,th	thermal
temp	Temperature
tu	turbine

## CRediT authorship contribution statement

**Armel Zambou Kenfack:** Writing – review & editing, Writing – original draft, Visualization, Validation, Supervision, Software, Resources, Project administration, Methodology, Investigation, Funding acquisition, Formal analysis, Data curation, Conceptualization. **Modeste Kameni Nematchoua:** Funding acquisition, Formal analysis, Conceptualization. **Elie Simo:** Funding acquisition, Formal analysis, Conceptualization. **Ghislain Junior Bangoup Ntegmi:** Investigation, Funding acquisition, Data curation, Conceptualization. **Venant Sorel Chara-Dackou:** Investigation, Funding acquisition, Formal analysis, Data curation.

## Declaration of competing interest

The authors declare that they have no known competing financial interests or personal relationships that could have appeared to influence the work reported in this paper.
